# Chronic Viral Liver Diseases: Approaching the Liver Using T Cell Receptor-Mediated Gene Technologies

**DOI:** 10.3390/cells9061471

**Published:** 2020-06-16

**Authors:** Katie Healy, Anna Pasetto, Michał J. Sobkowiak, Chai Fen Soon, Markus Cornberg, Soo Aleman, Margaret Sällberg Chen

**Affiliations:** 1 Department of Dental Medicine, Karolinska Institutet, 14152 Stockholm, Sweden; katie.healy@ki.se (K.H.); michal.sobkowiak@ki.se (M.J.S.); 2 Department of Laboratory Medicine, Karolinska Institutet, 14152 Stockholm, Sweden; anna.pasetto@ki.se; 3 Department of Gastroenterology, Hepatology and Endocrinology, Hannover Medical School, 30625 Hannover, Germany; Soon.Chai@mh-hannover.de (C.F.S.); Cornberg.Markus@mh-hannover.de (M.C.); 4 Cluster of Excellence RESIST (EXC 2155), Hannover Medical School, 30625 Hannover, Germany; 5 Centre for Individualized Infection Medicine (CiiM), 30625 Hannover, Germany; 6 Department of Medicine, Karolinska Institutet, 14152 Stockholm, Sweden; soo.aleman@ki.se

**Keywords:** chronic infection, viral hepatitis, T-cell immunotherapy

## Abstract

Chronic infection with viral hepatitis is a major risk factor for liver injury and hepatocellular carcinoma (HCC). One major contributing factor to the chronicity is the dysfunction of virus-specific T cell immunity. T cells engineered to express virus-specific T cell receptors (TCRs) may be a therapeutic option to improve host antiviral responses and have demonstrated clinical success against virus-associated tumours. This review aims to give an overview of TCRs identified from viral hepatitis research and discuss how translational lessons learned from cancer immunotherapy can be applied to the field. TCR isolation pipelines, liver homing signals, cell type options, as well as safety considerations will be discussed herein.

## 1. Introduction

### 1.1. Liver Tolerance and Implication for Diseases

The liver is a critical organ that performs an array of functions to support the body´s metabolism, immunity, digestion, detoxification, and vitamin storage. Located at the interface between the gut and the systemic circulation, it receives approximately 70% of its blood supply from the hepatic portal vein. As portal blood also transports a large number of foreign molecules (e.g., dietary antigens), the liver’s default immune status is essentially anti-inflammatory or immunotolerant, but it is also capable of mounting a rapid and robust immune response under appropriate conditions. The tolerogenic environment is evident in its low occurrence of post-transplant rejection, even when accepting a major histocompatibility complex (MHC) mismatched allograft [1]. While these immunosuppressive properties may prevent severe autoimmune responses and liver transplant rejections, they are disadvantageous against pathogens that have specialized in infecting the liver. An invading pathogen can induce a persistent inflammatory response which may lead to further liver complications such as the development of fibrosis, cirrhosis, and eventually hepatocellular carcinoma (HCC).

Host immune surveillance is critical for the detection and removal of aberrant and/or transformed cells and requires an effective crosstalk between the innate and adaptive immune systems. However, in cases of advanced liver disease, especially in HCC patients, dysfunctional T cell phenotypes are well described, and immune checkpoint molecules such as cytotoxic T-lymphocyte-associated protein 4 (CTLA-4) and programmed cell death protein 1 (PD-1) are upregulated in HCC-infiltrating T lymphocytes [2,3]. CTLA-4 negatively modulates the priming phase of the T cell response by outcompeting CD28 for binding of its ligands CD80/86, which are found on the surface of antigen-presenting cells (APCs), resulting in inhibitory signaling. CTLA-4 is upregulated in activated T cells and is also highly expressed on regulatory T cells (Tregs). PD-1 is another checkpoint molecule mainly found on mature peripheral T cells, natural killer (NK) cells, and several APCs. Its ligand, PD-L1, is highly expressed in HCC, and the constitutive activation of this axis leads to T cell exhaustion. Other inhibitory immune checkpoint molecules found to be upregulated in HCC include T cell immunoglobulin mucin-3 (TIM-3) and Lymphocyte-activation gene 3 (LAG-3) [3].

### 1.2. Chronic Viral Hepatitis

The major causative pathogens of chronic viral hepatitis are the hepatitis B, C, delta, and E viruses (HBV, HCV, HDV, and HEV, respectively). These are genetically and structurally distinct pathogens, although they share liver tropism and primarily infect hepatocytes. The replication cycle of these viruses is typically not directly cytopathic; instead, immune-mediated destruction of the infected cells, which is required for virus clearance, holds the significant contribution to liver injury and inflammation. Therefore, the antiviral immune response represents a double-edged sword that governs the clearance of infection, as well as disease progression. Within each virus family, there is varying heterogeneity at genotypic and strain levels that also influence host immunity, including mutations within immunodominant regions that contain B and T cell epitopes [4]. Several intrahepatic immune cells contribute to virus elimination, although virus-specific cytotoxic T lymphocytes (CTLs) are considered most central to successful viral clearance. CTLs exert both lytic and non-lytic effector functions on infected hepatocytes, and a polyfunctional antiviral T cell response is known to favor termination of the infection. Host failure to elicit a robust T cell response during early infection not only leads to chronic infection but is also accompanied by T cell anergy and deletion. Moreover, T cell exhaustion due to the upregulation of inhibitory co-receptors, inefficient priming by CD4^+^ T cells, suppressive signaling from regulatory Tregs, or the local cytokine milieu can all contribute to CTL failure during infection [5].

### 1.3. HBV

#### 1.3.1. Epidemiology and Virology

The global incidence of chronic HBV infection ranges from 240 to 350 million people affected. HBV is an enveloped DNA virus that replicates through an RNA intermediate and has features similar to retroviruses [6]. The virus is typically transmitted vertically from mother to child, or horizontally among adults through contaminated blood or body fluid. Exposure to HBV in children is associated with viral persistence in more than 90% of cases, while adults typically resolve infection in the acute phase. Upon infection, the viral particles attach to the hepatocytes where the viral nucleocapsid is injected into the cytoplasm and trafficked to the nucleus. Thereafter, a covalently closed circular DNA (cccDNA) and viral mRNA are generated, and viral transcription and assembly take place in the cytoplasm where new enveloped particles are released. The cccDNA is central to HBV persistence, even in patients where other serological HBV markers are absent [7].

Resolution of an acute self-limiting HBV infection is typically attributed to a robust, broad, polyfunctional T cell response of antigen-specific CD4^+^ and CD8^+^ T cells, as well as antibodies targeting HBV structural antigens [5]. Viral control is mediated by CD8^+^ T cells through non-cytolytic mechanisms, mainly by interferon (IFN)-γ and TNF secretion, and cytotoxic killing of infected hepatocytes [5,8,9]. Late, transient, narrow-range T cell responses are hallmarks of chronic HBV infection. Initial failure to control the virus leads to the constitutive activation of HBV-specific T cells which leads to an exhausted phenotype and terminal deletion [5,8,9,10]. Ex vivo selection and expansion are typically required to detect these T cells as their frequency reduces dramatically over the course of the disease [7].

During the HBV replication process, the viral genome is clonally inserted into the host chromosomal DNA [11,12]. The inserted HBV gene size ranges from 28 to 3215 bp, with some including the entire full-length HBV DNA sequence, and can result in intracellular DNA rearrangement, chromosomal instability, and increased mutagenesis. The location of the inserted viral sequence has been known to drive tumorigenesis by integrating within genes associated with cancer-related pathways such as the hedgehog signaling, extracellular matrix receptor, and mitogen-activated protein kinase genes [13]. The stable integration of HBV proteins in the host chromosome can result in a steady presentation of HBV-specific antigens on the surface of infected hepatocytes, making it an ideal candidate antigen for personalized therapies.

#### 1.3.2. Currently Available Treatments

Two types of treatments are available for chronic hepatitis B: pegylated interferons (IFNs) and nucleo(s)tide analogues (NAs). IFNs directly suppress viral RNA and protein production in infected hepatocytes, as well as modulate the host adaptive immune response, including HBV-specific T cells. NAs only inhibit HBV DNA replication and have no significant effect on immune responses or the cccDNA reservoir. Although NAs are not entirely capable of achieving HBV cure, they are capable of suppressing HBV replication without inhibiting antigen production. The latter has implications for immunotherapeutic targeting [14]. Unfortunately, none of the available HBV antivirals eliminate the risk of HCC development, and side effects and low response rates are also of concern.

### 1.4. HCV

#### 1.4.1. Epidemiology and Virology

Around 70% of HCV-infected patients become chronically infected with the risk of developing cirrhosis and HCC [15]. Globally, 71 million people are affected by chronic hepatitis C. One of the primary contributors to chronic HCV infection is the high rate of mutation of the virus, particularly in the envelope glycoprotein sequences [5]. While anti-HCV antibodies have been known to persist in chronic HCV, their capacity to neutralize the virus is limited due to the structural variability [5]. On the other hand, the cellular response mediated by CD4^+^ and CD8^+^ T cells is very important for viral control. The presence of HCV-specific CD4^+^ T cells during acute HCV infection is associated with the control of viral load and if sustained, also with HCV viral clearance [16]. The mechanism behind the development of chronic infection is associated with the impaired functionality of HCV-specific T cells, in particular, the HCV-core specific CD4^+^ T cell response appears to mediate a skewing in the T cell functionality, suppressing IL-2 production and increasing IL-4 and IL-10 secretion [17]. This results in ineffective viral clearance and a lack of support to CTL proliferation that complicates the elimination of virus-infected hepatocytes [5,10]. HCV-specific CTLs in chronically infected individuals are known to be ineffective and demonstrate an exhausted phenotype [8,9,18,19].

#### 1.4.2. Currently Available Treatments

The major revolution in HCV treatment has been the introduction of direct acting antivirals (DAAs). Before the emergence of DAAs, pegylated IFN-α and ribavirin were the mainstay of HCV treatment. Ribavirin improved the therapeutic efficacy of IFN-α by counteracting its immunosuppressive properties on NK and T cell functions [10]. HCV-specific CD8^+^ T cell proliferative potential at treatment baseline positively correlated to a rapid and sustained viral response (SVR), suggesting CTL effector cells can benefit therapeutic strategies [20]. Today, treatment with DAAs is effective, and sustained virological response is observed in more than 95% of patients. The impressive improvement in the clinical long-term outcome also reduces incidences of hepatic decompensation and HCC. Whether successful HCV cure by DAA is associated with normalization of innate immunity is currently a matter of debate [21,22], and so far, only transient and partial restoration of CD8^+^ T cells is reported [23].

### 1.5. HDV

#### 1.5.1. Epidemiology and Virology

HDV is a small defective RNA satellite virus that uses HBV surface antigen for propagation and the production of new virions. The clinical manifestations of HDV infection in HBV co-infected individuals are typically more severe with a higher incidence of HCC and increased liver-related and overall mortality. HDV infection is an understudied disease and as such, epidemiological data relating to its global prevalence varies widely, with recent studies estimating that somewhere between 12 and 72 million people have experienced the virus [24,25]. HDV replication occurs in the nucleus of hepatocytes, and it has been proposed that it replicates using a double rolling circle mechanism. The key to HDV assembly and release is hepatitis B surface antigen (HBsAg), which envelopes the virus, and infects through the sodium taurocholate co-transporting polypeptide (NTCP) receptor using mechanisms similar to HBV [26].

As with other hepatitis viruses, host failure to mount an effective CTL-mediated response to virally infected cells results in chronic HDV infection. CTL failure in HDV has been attributed to several factors, but a lack of CTL epitopes and the development of escape variants are important on a viral level. HDV-specific T cell responses are key for clearance but also mediate the large-scale tissue necrosis associated with the disease morbidities. HDV-specific CD4^+^ T cells release antiviral cytokines such as IL-10, IFN-γ, and IL-2, which in turn prime virus-specific CTLs and T helper 1 (Th1) cells. The infection severity has been attributed to an imbalance of type 1 and type 2 immune responses, as well as the mutation of immune escape variants with reduced human leukocyte antigen (HLA) binding [4,27,28].

#### 1.5.2. Currently Available Treatments

Currently, the only approved HDV treatment is pegylated IFNα, with poor clinical success rates of about 30% and severe associated side effects [26]. Clinical trials are ongoing with phase-3 studies being the most advanced, including the entry inhibitor bulevirtide, the nucleic acid polymer REP 2139-Ca, the farnesyltransferase inhibitor lonafarnib, and pegylated IFNλ [29].

### 1.6. HEV

#### 1.6.1. Epidemiology and Virology

Hepatitis E virus (HEV), a non-enveloped single-strand RNA virus is the most common cause of acute hepatitis in the world. Endemic HEV spread is usually through contaminated water, resulting in acute hepatitis infection, but it is resolved once neutralizing antibody is produced. In immunocompromised hosts, HEV genotype 3 often induces chronic hepatitis that progresses to liver fibrosis and cirrhosis. Recently, it has also been reported that HEV genotype 4 and HEV strains from the Orthohepevirus C species are also associated with persistent hepatitis in vulnerable patient groups [30,31]. Immunosuppressed transplanted patients are particularly vulnerable as the screening of blood products for HEV is not enforced in all countries. Patients with acute hepatitis E often spontaneously clear the virus. Immunosuppressed patients such as solid organ transplant patients are vulnerable and prone to develop chronic hepatitis E [32].

#### 1.6.2. Currently Available Treatments

There is currently no antiviral treatment available for chronic HEV, especially for those with chronic infection due to immunosuppression. When solid organ transplant patients are first diagnosed with HEV, a reduction in immunosuppressant should be the first course of action to allow spontaneous viral clearance. If this is unsuccessful, off-label ribavirin is used. Although ribavirin can achieve SVR in many cases, relapses do occur. Ribavirin is also reported to induce mutagenesis in HEV, leading to enhanced viral replication and eventually drug failure [33]. In cases of ribavirin failure, pegylated-IFNα is the last resort. Since it is associated with severe complications, possibly triggering organ rejection, pegylated-IFNα should only be used in liver transplant patients. For all other patients who failed ribavirin, no alternatives are available [34,35]. Although sofosbuvir, a DAA drug targeting HCV polymerase was found to be effective in limiting HEV viral replication in vitro [36], it failed to cure ribavirin resistant patients in vivo (ClinicalTrials.gov identifier NCT03282474).

### 1.7. Immunotherapy and Chronic Viral Hepatitis

Given the ability of immune control of viral hepatitis infection, immunotherapeutic strategies are a promising approach to achieving a cure. The use of immune checkpoint inhibitors (ICIs) acting on PD-1/PD-L1 and CTLA-4 have proven to be an exceptionally significant breakthrough in cancer immunotherapy. However, in HCC, so far, the result of these therapies has been limited mainly due to low response rates, which could be explained by the scarce distribution of tumor-infiltrating lymphocytes (TILs) in HCC [37].

To date, clinical trials investigating ICI in cancer-free viral hepatitis patients have specifically focused on anti-PD-1 therapy. In fact, a recent clinical study reported that the PD-1 inhibitor nivolumab in HBeAg-negative patients led to HBsAg decline in most patients and sustained HBsAg loss in one patient [38]. The administration of nivolumab was performed with and without an HBV-specific therapeutic yeast-derived vaccine, which was hypothesized to increase the frequency of vaccine-induced HBV-specific CTLs, but the addition of the vaccine had no significant benefit [38]. Another clinical study investigating PD-1 blockade in a chronically infected HCV cohort demonstrated an overall decrease in HCV replication, but only 12% of the participants achieved the primary endpoint of ≥ 0.5 log reduction in viral RNA levels [39]. In both studies, the ICI treatment was mostly well tolerated, while side effects were limited to grade 1to grade 2 immune related adverse events.

Adoptive cell transfer (ACT) of autologous ex vivo expanded T cells is a highly effective treatment for multiple cancer types and chronic viral infections. Lately, chimeric antigen receptor (CAR) and TCR-redirected T cells have taken ACT to the next level with their pre-designed antigen specificity. CARs are membrane-bound proteins comprised of an extracellular single-chain variable fragment (ScFv) of a monoclonal antibody (for antigen recognition), transmembrane domain, and intracellular signaling domain of the classical TCR complex. CARs recognize cell surface antigens in an HLA-independent manner and thus require a distinct tissue-restricted target antigen on the tumor cell surface. On the other hand, TCR-redirected T cells are based on classical TCR-mediated recognition and downstream signaling pathways. TCRs recognize epitopes derived from the entirety of the cellular proteome, forming a diverse range of antigen classes, including tissue-differentiation antigens, cancer germline antigens, viral oncoproteins, and mutated antigens (cancer neoantigens). Unlike CARs, the TCR targets components of the subcellular compartment and when expressed by CTLs, their ligand is most commonly an 8 to 11 amino acid linear peptide sequence presented in complex with host HLA molecules [40].

Both CAR-T cells with antibody-like specificity and TCR redirected T cells targeting HBV structural protein antigens have been shown to mediate the clearance of HBV-infected hepatocytes in vitro [41] and in animal models [42,43]. In mouse models, this approach reduces HBV replication and causes only transient liver damage. Moreover, CAR-T cells are functional regardless of HLA type but may also bind circulating virus decoys that interfere with target cell recognition; however, this was not seen in the preclinical mouse model (corresponding to the low-replicative phase of chronic hepatitis B) that was given retrovirally transduced CAR-T cells [42,43]. Overall, these data are encouraging in the perspectives of persistent viral diseases in the liver that are difficult to cure today.

## 2. Overview of Published CTL-Derived TCRs with Hepatitis Virus Specificity

### 2.1. TCR Reconstitution in Human T Cells and Clinical Evidence

Some of the earliest clinical studies investigating the feasibility and safety profile of ACT were performed in patients with viral infections such as Epstein–Barr Virus (EBV) and cytomegalovirus (CMV) [44]. Due to advancements in the development of antiviral drugs, the broad application of ACT to treat viral infections has been limited, except for immunocompromised patients and/or those with end-stage disease. Patients who have undergone allogeneic hematopoietic cell transplantation (HCT) are at particular risk of acquiring viral infections, which are associated with an increase of morbidity and mortality [45]. An example of the success of ACT for this patient group is the study by Pei et al. [46], where HCT patients with refractory CMV received an infusion of CMV-specific T cells isolated from healthy donors. Within 4 weeks, 27 of the 32 patients cleared the infection without recurrence [45].

Lately, several TCRs from antiviral CTLs to various human hepatitis viruses have been identified, which have been functionally characterized using peripheral T cells expanded from respective patient groups and healthy blood donors in experimental studies (Table 1). To date, clinical studies using TCR-redirected T cells targeting viral hepatitis antigens have focused on the treatment of hepatitis-related malignancies, of which HBV-related HCC is the best characterized. As a result of its chromosomal integrative abilities, most HCCs that arise as a result of chronic HBV infection contain short HBV DNA fragments, which encode potential epitopes for redirected T cells [47]. At the time of writing, there are currently three studies in phase 1 clinical trials that utilize HBV peptides as tumor-associated antigens (TAAs), either as prophylaxis (NCT02686372) or treatment for recurrent HCC post-transplantation (NCT02719782) or liver resection (NCT03899415).

TCR-T cell therapy targeting HBV antigens has been demonstrated in the treatment of a chronic HBV patient with extensive HCC metastases following liver transplantation [58]. Since most HBV-infected hepatocytes were removed as a result of the transplant, the potential for T cell-mediated destruction of non-tumor tissues following infusion of the T cells was minimized. Furthermore, immunohistochemical analysis of the metastasized lesions showed that they expressed the TCR target, HBsAg, which was also used as a marker to monitor the efficacy of the treatment. Transplanted T cells expanded well in vivo, without any detectable side effects, and resulted in a 90% reduction in serum HBsAg levels over a 30-day period [58].

Technically, TCR-redirected T cell therapy for liver viral diseases has entered an exciting time, with a growing number of described epitopes and corresponding TCRs. Important considerations for their clinical translation are balancing the therapeutic benefits with safety, particularly given the inflamed status of the chronically infected liver. The ideal T cell therapy would reduce the viral and antigen burden and boost local antiviral responses while minimizing side effects such as hepatic flare. Interpatient variation in the extent of hepatocytes that are presenting viral antigens on their surface has implications for such adverse events and can be dependent on viral strain and stage of infection [5,59,60]. Genotypic variations and mutational susceptibility in virus proteins may affect efficient TCR recognition, and analysis of biopsy samples and circulating antigens will prove useful for treatment compatibility.

### 2.2. Selection of Viral Epitopes

The rationale of viral hepatitis-specific immunotherapy is heavily dependent on the precise recognition of the candidate viral epitopes by the TCRs; therefore, a main concern would be viral epitope escape, driven by the immunological pressure. Unlike natural virus-specific T cells, which are polyclonal and can be expanded ex vivo, genetic engineering of T cells with epitope-specific TCRs augments the pressures of immune escape. Therefore, ideal epitope candidates should be stable and highly conserved viral sequences, preferably within which viral fitness is locked [61]. For RNA viruses that are more prone to mutation, viral fitness limits variability within immunological epitopes; thus, functional helicases and RNA-dependent RNA polymerase (RdRP) complexes are attractive CTL targets. As reported for HCV, immunodominant viral helicase NS3_1073_-specific and NS3_1406_-specific T cell populations are frequently observed in patients with resolved infection. Characterized by strong cytolytic and proinflammatory cytokine responses, particularly in terms of IFN-γ production, they are noted of being of high-avidity and preferentially control the viral infection [62]. TCR redirection of human T cells toward these antigens renders them responsive to HCV-bearing hepatocytes and inhibits viral replication in vitro, proving their potential in TCR therapy [53,54,55]. In Soon et al. [57], TCRs targeting epitopes at HEV RdRp and helicase domains were suggested as immunotherapeutics for immunosuppressed patients. Although these epitopes are functional as shown in an independent study by Brown et al. [63], whether they are dominant in immunocompetent patients remains to be elucidated. Preclinical testing in well-established human liver reconstituted animal models should provide more insights and allow better evaluations of the epitopes of choice.

### 2.3. Factors Impacting Specificity and Safety

#### 2.3.1. Gene Transfer Methods

Stable TCR integration is desirable for achieving persistent antigen-specific T cells in vivo, but to date, insertional mutagenesis or uncontrolled proliferation is still of regulatory concern. For essential organs such as the liver, where a significant proportion of the total hepatocyte population could be occupied by infected cells, the risk of prolonged excessive immune-mediated damage is a serious risk. Transient expression of TCR genes is one alternative by which these risks can be mitigated. In vitro transcribed TCR mRNA redirected T cells express the transferred TCR genes for several days. Although they display less cytotoxic potential when compared to virally transduced T cells in vitro, [64,65,66], they have proven to be safe and effective in a clinical trial involving a chronic HBV patient with extensive HCC metastasis [58]. Administering multiple escalating infusions of mRNA TCR-redirected T cells specific for HBV antigens resulted in reduction of the metastases, which could not be explained entirely by the direct lytic effect of the administered T cells, but rather by their induction of a secondary antitumor response. Furthermore, unlike some transduction methods that require the pre-activation of T cells, it is possible to use resting T cells [66] that possess lower levels of perforin and granzymes but retain antiviral mechanisms. The latter could also be achieved by lowering the TCR functional avidity [54] to reduce the risk of excessive hepatoxicity.

#### 2.3.2. TCR Cross-Reactivity

Cross-reactive TCRs in therapy could trigger undesirable off-target toxicity, such as those observed in a melanoma-associated antigen (MAGE)-specific TCR immunotherapy, as these epitopes are originally derived from self-peptides and could still be expressed by normal cells, in this case those found in the cardiac tissue [67]. Recently, a HEV_1527_-specific TCR was found to cross-recognize an apoptosis-related epitope: Non-muscle myosin heavy chain 9 (MYH9-478: QLFNHTMFI), as a unique multiple glycine motif, was noted in the TCRβ CDR3. However, further investigation showed that the TCR only recognized but was not reactive toward the self-antigen [57]. While this is an important reason why mutated cancer neoantigens are strongly preferred, it is sensible to consider cross-reactivity studies in preclinical evaluations of new TCR candidates.

#### 2.3.3. TCR Mispairing

Potential mispairing with endogenous α and β chains may create unpredictable specificity and decrease the expression of transferred TCR genes. To overcome this drawback, several strategies have been developed [68]. For example, one approach is murine or murinized TCRs, which are not capable of recombination with endogenous human TCRs [52,69]. Other methods include modification of the TCR constant chain, introduction of a second sulfide bond between the TCRα and β chains, and the use of the 2A self cleavage peptide between the TCRα and TCRβ chain in the same plasmid [68]. Advancements in gene editing tools also enable the simultaneous knock-out of expression of the endogenous TCR at the same time as the transduction of the new genes using CRISPR/Cas9 technology [70]. Another benefit of this approach is the possibility of using it to generate universal therapies, as clinically demonstrated using transcription activator-like effector nuclease (TALEN)-edited CAR-T cells [71].

## 3. Expanding the Repertoire: Improved Approaches to TCR Discovery

To date, most of the techniques used to identify TCRs for immunotherapy have primarily been performed when investigating tumor-reactive TCRs from the TIL populations of cancer patients. Identification of virus-specific TCRs can also be performed by adapting currently available protocols.

### 3.1. Enrichment Markers for Antigen Reactive T Cells

Persistently antigen-exposed effector T cells express inhibitory and exhaustion markers such as PD-1, TIM-3, and LAG-3. Among these markers, only PD-1 has been used along with the relative TCRβ clonotype frequency to identify candidate tumor-reactive TCRs [72,73]. Among integrins, CD103 on a CD8^+^ TIL subset in solid tumor correlates to favorable prognosis, and its co-expression with CD39 has been proposed as a marker for the identification of tumor-reactive CD8^+^ TIL [74]. Other markers that have been proposed for the identification of neoantigen and tumor-reactive T cells are activation markers such as CD137, directly ex vivo [75,76] or after stimulation with mutated peptides and minigenes expressing neoantigens [77]. In HBV, the increased expression of T cell memory markers such as CD127, CD27, CD45RO, and CXCR3, coupled with the decreased expression of CD57, PD-1, and TIGIT on HBV_core169_-specific CTLs has been linked to viral control [78]. Although this type of strategy allows for the enrichment of an antigen-reactive bulk population, each individual TCR still needs to be evaluated independently to confirm specificity.

### 3.2. MHC-Multimer Guided Isolation of Virus-Specific T Cells

A number of new technical advancements has recently made it possible to isolate a large number of TCRs based on their ability to bind to MHC multimers loaded with a defined minimal epitope [79,80,81]. The generation of MHC-tetramer panels comprised of viral epitopes, in combination with the most common mutations observed within them, represents a good source for isolating virus-specific TCRs from peripheral blood. A useful advantage of this epitope-specific approach is that virus-specific TCRs can also be isolated from healthy donors that may have already resolved the infection, and it has successfully been used for the discovery for HEV-specific TCRs [57]. Some limitations of this strategy include the isolation of antigen-specific T cells regardless of functional status, the availability of well-defined peptide epitopes, and a lack of MHC multimers to many class I and class II restrictions [82,83,84].

### 3.3. T Cell Bulk Sequencing and Pairing by Frequency

Next-generation sequencing (NGS) technology has been implemented to characterize the immune receptor repertoire from a variety of sample types and T cell populations. TCR-NGS is usually targeted for the highly variable CDR3 region that can be used as a molecular ID for a specific TCR clonotype. The possibility to sequence at high read depth also allows the detection of very low frequency clonotypes in a variety of different sample types in healthy individuals [85] or after allogeneic HCT [86], for example. One major limitation of this approach is that it does not identify the matching TCRα and TCRβ chain from a polyclonal population; it can only provide the relative frequencies of the two chains in the sample studied. In oligoclonal T cell samples, where the relative frequency of the most dominant clonotype is expected to be more than 20%, it is possible to successfully pair the most dominant TCRβ clonotype with the most dominant TCRα clonotype and obtain functional, antigen-reactive TCRs [73,77]. Another method that has been utilized to match TCRα and TCRβ chains from a bulk T cell population is the Pairseq platform [87]. This approach is based on NGS of both TCRα and TCRβ chains and pairing of the two chains is assigned with a statistical algorithm.

### 3.4. Single Cell Sequencing

Ideally, this represents the best approach because it enables the correct TCRα–TCRβ pairs from each single cell present in the T cell population of interest. Several different techniques have been developed to achieve this goal; for most of them, the throughput is still in the order of a few hundreds of cells at a time, but new high-throughput technologies have now been recently introduced on the market with promising results.

Several platforms have been developed to allow the construction and the NGS of full mRNA transcripts obtained by one cell. This method not only provides full-length TCR sequences for both chains at the same time but can also provide information on other transcripts (for example cytokines) expressed by the T cells. One application of the single cell RNA-sequencing performed with the Fluidigm platform has been recently described for the identification of tumor-reactive TIL [88]. In this study, the TCR paired sequences were identified from cells that were also expressing high levels of IFN-γ and IL-2 after co-culture with tandem minigene-transfected or peptide-pulsed autologous patient APCs. The major limitation of this method is the cell number required to perform the assay, which can be an issue when screening T cell populations where the reactive clonotypes are present at low frequency as in chronic viral hepatitis and cancer [64].

Single cell RT-PCR used in combination with flow cytometric cell sorting offers the possibility to isolate and sequence T cells based on a combination of surface markers. The amplification of TCR genes is often carried out by gene-specific primer cocktails, and the sequencing of the PCR product could be done by traditional Sanger or NGS [81,89]. One limitation of this approach is that the throughput is still relatively low, and additional primers are needed to provide a functional characterization of the T cells.

Limiting dilution T cell cloning provides a highly sensitive and specific platform to isolate clinically relevant antigen-reactive T cells or their TCRs for viral treatment. It is based on the enrichment of T cells expressing PD-1 and/or T-cell activation markers such as CD134 and CD137, followed by microwell culturing at limiting dilution cell concentrations to avoid the overgrowth of non-reactive T cells. This strategy successfully led to the detection of CD4^+^ and CD8^+^ T cells targeting 19 neoantigens compared to only 8 neoantigens recognized using the unbiased TIL screening approach [90].

Emulsion-based PCR technologies allow the identification of TCRα–TCRβ pairs from single cells that are contained within oil droplets [91]. Further technical developments have recently been achieved with the 10X Genomics platform that allows higher throughput (up to hundreds of thousands of cells simultaneously) for both TCR sequencing and other cell transcripts.

## 4. Toward Liver Homing: Unconventional T Cells

### 4.1. Physical, Metabolic, and Immunological Constraints in the Liver Microenvironment

Key to the efficacy of TCR-redirected T cell therapy is the ability of the cells to migrate from the blood to the target tissue and adapt to their local environment. Due to their accessibility, peripheral conventional T cells are clearly the most straightforward choice for T cell therapies. However, once they reach the liver, there will be physical, metabolic, and immunological barriers that may dampen their efficacy. Immunosuppression from potent regulators within the liver milieu (Tregs, granulocytic myeloid-derived suppressor cells, TNF-related apoptosis-inducing ligand (TRAIL)-expressing NK cells) [92], immunometabolic constraints on virus-specific T cells (hypoxia [65], inability to use oxidative phosphorylation [92]), and competition for local nutrients (low arginine [92,93]) are all potential mechanisms that can inhibit adoptively transferred T cells.

These limitations need to be considered during therapy design, and rigorous preclinical evaluations have already been established to model these challenges. Lately, the development of human liver organoids [94], 3D cell culture modelling systems [65], and in vitro infection models of primary cells [94] has enabled interrogative studies of the challenges faced by hepatotropic T cells ex vivo. Proof-of-concept was demonstrated by Pavesi et al. [65], when investigating the impact of local factors on the function and tissue migration of TCR-redirected T cells in a liver-like microenvironment. Challenges such as low oxygen and the presence of inflammation were tested in a 3D microfluidics system engrafted with HBV antigen-expressing target cells. They revealed that mRNA TCR redirected T cells possessed a lower cytolytic capacity when compared with retrovirally transduced T cells, which could not be seen in a classical 2D culture, where cell motility cannot be evaluated. Furthermore, the introduction of monocytes into the system showed that retrovirally transduced TCR-redirected T cells, but not mRNA TCR-redirected T cells, were sensitive to immunosuppression through the PD-1/PD-L1 axis [95]. The customizable nature of these types of models will not only bring in vitro studies closer to more physiologically relevant conditions but can also provide predictions on the likely success and failure of new approaches before their clinical translation.

One such strategy to benefit the outcome of adoptively transferred T cells may be to exploit the characteristics of the T cells that naturally reside in the liver. The redirection of T cells with inherent hepatotropism may result in enhanced migratory and functional abilities when compared with conventional T cells from the periphery, which are currently in clinical use.

### 4.2. Liver Resident T Cells and Unconventional T Cell Subsets

Peripheral tissues, in their role as barriers to the outside world, require a mechanism to both provide a rapid immune response against new infection, as well as maintain barrier homeostasis in the presence of a variety of microbial and environmental hazards. That role is served by tissue-resident immune cells, a category encompassing a wide variety of cell types, such as NK cells, dendritic cells (DCs), macrophages and innate lymphoid cells (ILCs) [96,97,98,99], and importantly for this review, T lymphocytes [100]. Of particular interest are CD8^+^ tissue-resident memory T (T_RM_) cells, which establish a non-recirculating sentinel population in peripheral tissues following infection [101]. Unconventional T cell types have also been found to share a tissue-resident phenotype, such as natural killer T (NKT) cells [102], mucosa-associated invariant T (MAIT) cells [103,104], and γδT cells [105]. Among the various tissues in which tissue-resident T lymphocytes were identified, the liver plays a prominent role [106]. Several studies have delved deeper into the liver T_RM_ cell population [106,107,108,109,110], and it has been shown that liver MAIT cells also show hallmarks of a tissue resident phenotype [103].

Characteristic markers of tissue resident lymphocytes across various tissues are CD69, which prevents recirculation into the blood through the negative regulation of sphingosine-1-phosphate receptor 1 (S1P1) [111] and CD103 (integrin αEβ7), which binds E-cadherin [100], coupled with the expression of transcription factor Hobit/Blimp-1 [106]. Interestingly, only a small subset of CD8^+^ T_RM_ cells in the murine liver was initially reported to express CD103 [106,108], but Pallett et al. showed that in humans, while widely varying between donors, CD69^+^CD103^+^ T_RM_ cells can account for over 30% of non-naïve lymphocytes in the liver [110]. Human liver CD69^+^CD103^+^CD8^+^ T_RM_ exhibit hepatotropic virus specificity and elicit their effector functions in response to hypoxia-inducible factor (HIF)-2α, which is a transcription factor upregulated during liver pathogenesis [112]. However, as shown by McNamara et al., in the murine liver it is not CD103, but another marker, LFA-1 (lymphocyte function-associated antigen 1), which is critical to establishing tissue-resident memory T cell populations [109]. Similarly, LFA-1 is necessary for establishing murine liver-resident NKT cell populations [113], and it is also expressed by human liver MAIT cells [103]. LFA-1 binds ICAM-1 (intercellular adhesion molecule-1), and LFA-1–ICAM-1 interaction has long been identified as critical for T cell infiltration into the liver in hepatitis B infection [114,115]. Recent research in mice has also revealed a T_RM_ cell population necessary for immune response against liver-stage malaria [108], and that LFA-1–ICAM-1 interaction is critical to mounting said response [109]. A variety of liver-homing markers have also been shown to be necessary in protection against liver disease [116,117]. Such developments highlight the central role tissue-resident T lymphocytes play in liver pathology.

### 4.3. Redirection of Unconventional T Cells for Immunotherapy: What is Known

As summarized in Table 2**,** various subsets of unconventional T cells can be enriched at rather high numbers, and they have garnered much interest in cancer immunotherapy [118,119]. Among the three major subtypes, MAIT cells, and γδT cells represent the major T cell populations in the liver, with some studies showing that each of those subsets can comprise up to around half of all T lymphocytes in healthy human liver [103,120,121]. iNKT cells are a modest subset comprising around 0.5% of the total liver T cell content [122]. Although the majority of studies involving unconventional T cell immunotherapy have focused on modification with CARs, the availability of ex vivo expansion protocols and gene modification methods may expand their use to TCR redirection in the future.

#### 4.3.1. iNKT Cells

iNKT cells or Type I NKT cells are characterized by the expression of CD161 and NKG2D, as well as an invariant Vα24-Jα18-Vβ11 TCR. Their natural ligands are glycolipids such as alpha-Galactosylceramide (α-GalCer) and lipids released in response to intracellular infection [118]. iNKT cells can mount a TCR-dependent response against a wide variety of infectious agents and disease states. The potent cytotoxic capacity of iNKT cells, as well as their ability to recognize tumor cells, led to their recognition as potential vectors for cancer immunotherapy [118]. First clinical trials treated advanced solid tumors by intravenous α-GalCer injection [123]. Since then, strategies based on the ex vivo expansion of patient-derived primary iNKT cells entered several clinical trials over the years [124,125,126,127,128,129] that resulted in satisfactory safety data. They also came timely for the CAR-NKT cell platform for cancer immunotherapy. The first attempt was reported by Heczey et al. [130], who transfected primary human iNKT cells with CAR against GD2 ganglioside including 4-1BB (GD2.CAR). The first clinical trial of the CAR–NKT technology against neuroblastoma in children (NCT03294954) began in 2018 and await the first results in 2021. While that series of studies has so far been the only example of CAR–NKT generation culminating in a clinical trial, other CAR–NKT lines are being reported [131]. Notably, when the successful use of in vivo generated human CAR-T cells for tumor treatment in mice was recently demonstrated, the in vivo transfected cells included NKT cells alongside NK and conventional CD8^+^ T cells [132].

#### 4.3.2. γδT Cells

Unlike iNKT and MAIT cells, γδT cells exhibit some variance in their TCR antigen and exhibit a mix of adaptive and innate-like responses [105]. γδT cells do not require MHC antigen presentation for activation and primarily recognize phosphoantigens and antigens presented by several nonclassical MHC-related molecules, including CD1d and MR1. Clinical studies on γδT cells for cancer immunotherapy have mainly focused on the γ9δ2 subset, which can be efficiently expanded from PBMCs using zoledronate [133,134]. One strategy is focused on phosphoantigens or pamidronate/zoledronate paired with IL-2 to stimulate γδT cell activation and expansion in vivo. Another utilized ex vivo expanded PBMC-derived γδT cells stimulated with phosphoantigens or zoledronate + IL-2 [135,136,137,138,139,140,141,142,143,144,145]. Regardless of strategy, results were limited, although no trial reported adverse effects.

Some attention has been paid to the prospect of employing CAR-γδT cells for cancer immunotherapy. Efficient ex vivo expansion of γ9δ2 renders them good candidates for CAR strategies, and several CAR transfection protocols have been developed [146,147]. Notably, CAR-γδT cells exhibit similar antitumor activity to conventional αβ CAR-T cells while posing less risk for adverse side effects [147]. A clinical trial (NCT02656147) is currently ongoing using CD19 CAR-γδT cells against B-cell lymphoma, ALL (acute lymphocytic leukemia), and CLL (chronic lymphocytic leukemia) with completion expected in 2020.

#### 4.3.3. MAIT Cells

MAIT cells have a semi-invariant TCR of a Vα7.2 segment with either Jα12, 20 or 33, and a limited β chain diversity. Vitamin B2 metabolites presented by MR1 are the classical activating ligand of MAIT cells [148], but they can also be activated in an MR1-independent and/or cytokine-driven manner in response to viral infection [149]. These characteristics allow MAIT cells to respond to a broad range of pathogens in a rapid, innate-like manner. When activated, MAIT cells release a variety of cytokines, in particular TNF and IFNγ, as well as IL-17 and IL-22 for MAIT cells residing within tissues [148].

Interestingly, MAIT cells are a major subset of T cells in the liver, comprising up to 45% of total intrahepatic CD3^+^ lymphocytes, while only accounting for up to 8% of blood T cells, and they are specifically enriched within the bile duct [103,120]. MAIT cells’ high proportion within the human T lymphocyte population, as well as a number of similarities to iNKT cells, has led to their recognition as a potential platform for cancer immunotherapy, bypassing issues stemming from low human iNKT cell count, which marred iNKT cell immunotherapy [119]. MAIT cells infiltrate various mucosal tumors, although usually losing much of their functionality, particularly with regard to IFNγ production [150,151]. Although, cytotoxic functions appear to be retained in tumor-infiltrating MAIT cells [152]. However, the paradigm of MAIT cells as contributors to antitumor immunity has been challenged by the discoveries that colorectal cancer infiltration by MAIT cells correlates with poor prognosis [153] and that MAIT cells infiltrating HCC tissues are reprogrammed toward a tumor-promoting phenotype [154]. As a result of their low cellular frequency in mice, better experimental models than conventional mouse models will be required to evaluate their in vivo efficacy.

### 4.4. Future Outlooks

The use of hepatotropic T cell subsets warrants further investigation. As an emerging field of research, there is a lack of available safety data that justifies their selection over conventional T cell therapies. The precise conditions under which unconventional T cells are involved in pro- or anti-tumorigenic responses in the liver remains unclear and will need to be elucidated before their use as ACT therapies. In the meantime, it may be possible to look to the beneficial properties of these cell types and use them to optimize current treatments with conventional T cells. The introduction of liver-specific chemokine receptors and recapitulating the metabolic fitness of intrahepatic T cells are perhaps just some of the improvements that can be made to currently available products.

## 5. Concluding Remarks

As the field of TCR-redirected T cell therapy as a cancer treatment rapidly evolves, its application to chronic infection management is also likely to be feasible in the near future. Accumulating results from HCC immunotherapy studies have generated encouraging data. Using ACT to reconstitute virus-specific T cells for combating chronic infections of the liver may be realistic in situations where antivirals are ineffective. The discovery of more functional TCRs and careful evaluations in preclinical and clinical platforms will favor more safety and broaden therapeutic applications. Factors that remain difficult to assess are the influence of the stage of disease (persistent infection, primary tumour versus metastasis), where evaluations with preclinical animal models will give more insights. Therefore, current clinical trials involving CAR-T and TCR-based therapies targeting neo- and viral antigens within the liver will provide an invaluable indicator for the future clinical accessibilities of this therapy.

## Figures and Tables

**Table 1:** Hepatitis virus-specific T cell receptors (TCRs) and their targets. HBV, HCV, and HEV: hepatitis B, C, and E viruses.

Virus	Antigen	Amino Acid Position	Peptide Sequence(s)	HLA	Clinical Usage	Reference
HBV	HBV envelope	HBVS20-28	FLLTRILTI, FLLTKILTI	A201	N	[48]
	HBV envelope	HBVS172-180	WLSLLVPFV, WLSLLVQFV, FLGPLLVLQA	A201 Cw0801	N N	[48] [49]
	HBV envelope	HBVS183-191	FLLTRILTI	A201	NCT03634683	[50,51]
	HBV envelope	HBVS370-379	SIVSPFIPLL	A201	N	[49]
	HBV core	HBVC18-27	FLPSDFFPSV, FLPSDFFPSI	A201	N	[48,50,51]
HCV	HCV non-structural protein 3	NS31073–1081	CINGVCWTV	A201	N	[52,53]
	HCV non-structural protein 5	NS51992–2000	VLTDFKTWL	A201	N	[54,55]
HEV	RNA helicase RNA-dependent RNA polymerase	HEV1116-1124 HEV1527−1535	SLFWNEPAI LLWNTVWNM	A201 A201	N N	[56,57]

N: No data available.

**Table 2:** Summary of characteristics of unconventional T cell subsets. IFN: interferon, iNKT: invariant natural killer T cells, MHC: major histocompatibility complex, TCR: T cell receptors.

Cell Type	MHC Molecule	Amount in Healthy Liver (% of CD3^+^)	Functional Capacity	Liver Homing Markers Expressed	Involvement in Viral Hepatitis Response	Ex vivo Expansion Protocols	Examples of Animal Studies	Examples of Clinical Studies
iNKT	CD1d	≈0.5% [122]	IFNγ, TNF, IL-4, IL-13, IL-17, IL-21, IL-22, GM-CSF [155] High cytotoxic activity [156]	CXCR6, LFA-1, CD49a [113,157,158]	HBV: Involved in acute response; depleted in chronic infection; critical to viral control in chronic infection; activation in chronic infection contributes to cirrhosis development [159,160,161,162,163] HCV: Depletion in blood of HCV patients seen in some studies, but contradicted by others; activation in chronic infection contributes to cirrhosis development [159,164,165,166] HEV: Activated and depleted in the blood [167]	[124,128,168,169]	[170,171]	[123,124,125,126,127,128,129,172,173,174,175,176,177,178,179,180]
γδT cells	MHC class Ib, CD1c, CD1d, MR1 [105,181,182]	5–55% [121]	IFNγ, TNF, IL-4, IL-5, IL-6, IL-8, IL-10, IL-13, IL-17, IL-22, GrzB, GM-CSF [183] Cytotoxic capacity [183,184]	LFA-1, CXCR6, CXCR3 [121,185]	HBV: Highly activated, cytotoxic and infiltrating into liver in acute infection; Vδ2 subtype associated with chronic infection [186,187,188] HCV: Enriched and exhibiting high cytotoxic activity in liver; biased toward Vδ1 subset; heterogenous role depending on subset [183,189] HEV: Activation of Vδ2 subset in acute infection [190]	[133,134,146,147,191]	[192,193,194]	[135,136,137,138,139,140,141,142,143,144,145,195,196,197,198,199,200,201]
MAIT	MR1 [148]	Up to 45% [103,120]	IFNγ, TNF, IL-17, IL-22, GrzB [148] Cytotoxic capacity [148]	CCR5, CXCR6, CCR6, CXCR3, LFA-1, VLA-4 [103]	Functionally impaired in the blood and depleted in HBV, HCV, and HDV [120,202,203] Respond to HCV-infected cells in a TCR-independent, cytokine-driven manner [149]	[204]	[205]	N
